# Physicochemical, potential nutritional, antioxidant and health properties of sesame seed oil: a review

**DOI:** 10.3389/fnut.2023.1127926

**Published:** 2023-06-01

**Authors:** Edwige Bahanla Oboulbiga, Zoénabo Douamba, Diarra Compaoré-Sérémé, Judith Nomwendé Semporé, Rasmata Dabo, Zénabou Semde, Fidèle Wend-Bénédo Tapsoba, Fatoumata Hama-Ba, Laurencia T. Songré-Ouattara, Charles Parkouda, Mamoudou H. Dicko

**Affiliations:** ^1^Food Technology Department (DTA), Institute for Research in Applied Sciences and Technologies (IRSAT), National Center for Scientific and Technological Research (CNRST), Ouagadougou, Burkina Faso; ^2^Laboratory of Biochemistry, Biotechnology, Food Technology and Nutrition (LABIOTAN), Department of Biochemistry and Microbiology, University Joseph KI ZERBO, Ouagadougou, Burkina Faso

**Keywords:** sesame seed oil, sesame, nutritional value, polyunsaturated fatty acid, antioxidant capacity, health

## Abstract

Sesame (*Sesanum indicum* L.) is one of the primary annual oilseeds grown in Africa and Asia. Sesame seed oil (SSO) is of great economic and human nutrition interest worldwide. Due to its composition in phytochemical antioxidants and profile in unsaturated fatty acids, sesame is used as a biological source of essential fatty acids. It contains bioactive compounds such as lignans (sesamin, sesamol, sesamolin), tocopherols and phytosterols. The oleic/linoleic fatty acids ratio of sesame makes it important for human health. SSO has bioactive compounds that can help prevent certain cardiovascular, metabolized and coronary diseases. The ω-3 and ω-6 fatty acids in SSO are precursors to eicosanoids that regulate the immune system and inflammatory functions. The essential fatty acids contained in this oil are essential for cell construction and highly recommended during the first trimester of pregnancy. The consumption of SSO allows both a decrease in the LDL-cholesterol complex and an increase in the HDL-cholesterol complex. It regulates blood sugar and may have favorable effects on people with liver cancer and those developing fatty liver disease. In this review, the nutritional value, antioxidant properties, and health benefits of SSO have been compiled to provide collective information of nutritional and medical interest.

## 1. Introduction

Oil seeds occupy an important place, alongside cereals, in human nutrition and world trade. Their derived-products, in different commercial forms, contribute to food balance with a ratio of about 1/3 along with carbohydrates and proteins.

*Sesanum indicum* L. (sesame) is an oleaginous plant grown in China, India, Sudan, Japan, Mexico and many countries in West and Central Africa, and Central America. The increased interest for sesame nutritional value has led to a sharp increase in its consumption and use in confectionery. This change in consumption habits is reflected by the increasingly important use of sesame seeds in food products at the domestic and industrial levels (1, 2).

Sesame seeds, considered both as spice and oil seed, are balanced oil source. It has a higher oil content (about 50%) than most other well-known oil seeds (3, 4). Sesame seed oil (SSO) is among the most expensive and coveted edible oils in the world. It is, one of the healthiest oils containing 38.84% of oleic acid and 46.26% of linoleic acids that are high levels of unsaturated fatty acids, justifying SSO the classification of its oils in the group of oleic-linoleic acid containing oils (5). The first stabilizes cholesterol levels while the second allows the reduction of cholesterol levels, as well as the risk of cardiovascular accidents. SSO has antilipolytic effects in the body and can prevent the oxidation of low-density lipoprotein (LDL) complex with cholesterol. Its cholesterol-lowering activity is linked to phytoestrogen which increases high-density lipoprotein (HDL) and lowers LDL, VLDL (Very-low-density lipoprotein), TC (Total Cholesterol) and TG (Triglycerides) (3).

Moreover, a higher content of phytoestrogen and lignin in sesame seeds oil can significantly impact the oxidative stress and lipid profile (3). In addition, lignans such as sesamin, sesamol and sesamolin, and phytosterols are found in SSO. Their antioxidant properties may explain both the superior stability of the oil and its beneficial health effects (6). Sesamin and sesamolin the two main lignans detected in SSO, have numerous health benefits, such as anti-inflammatory, antioxidant, hypocholesterolemia, neuroprotective and antihypertensive activities (7). Additionally, it is reputed to be highly oxidatively stable due to its richness in vitamin E (α-, β-, γ-, and δ-tocopherols and the corresponding four tocotrienols homologs) and is also widely used in margarine (8).

This review article summarizes the literature related to the nutritional value of SSO, its antioxidant properties and health benefits. The keywords in literature review were: SSO, sesame seed, sesame, nutritional composition of SSO, antioxidants of SSO, benefits of SSO, comparison of SSO with other oils. These keywords were collected from Google Scholar, PubMed, Scopus, Web of Science databases published in between 1990 and 2022. The literature search yielded a total of 938 results. Studies on the physicochemical composition of SSO and sesame grains, production of SSO and sesame seed, extraction of SSO, benefits of SSO, comparison of SSO with other oils, Vitamin E, sesamin, sesamol and sesamolin were included in this review. The titles and abstracts of the papers obtained were evaluated against the objectives and scope of the review. Duplicates were rejected. Letters, comments, communications were also excluded. A total of 70 studies were included in this article.

## 2. Global sesame seeds production overview and trade and sesame oil

### 2.1. World sesame seed production

**Figure 1:**
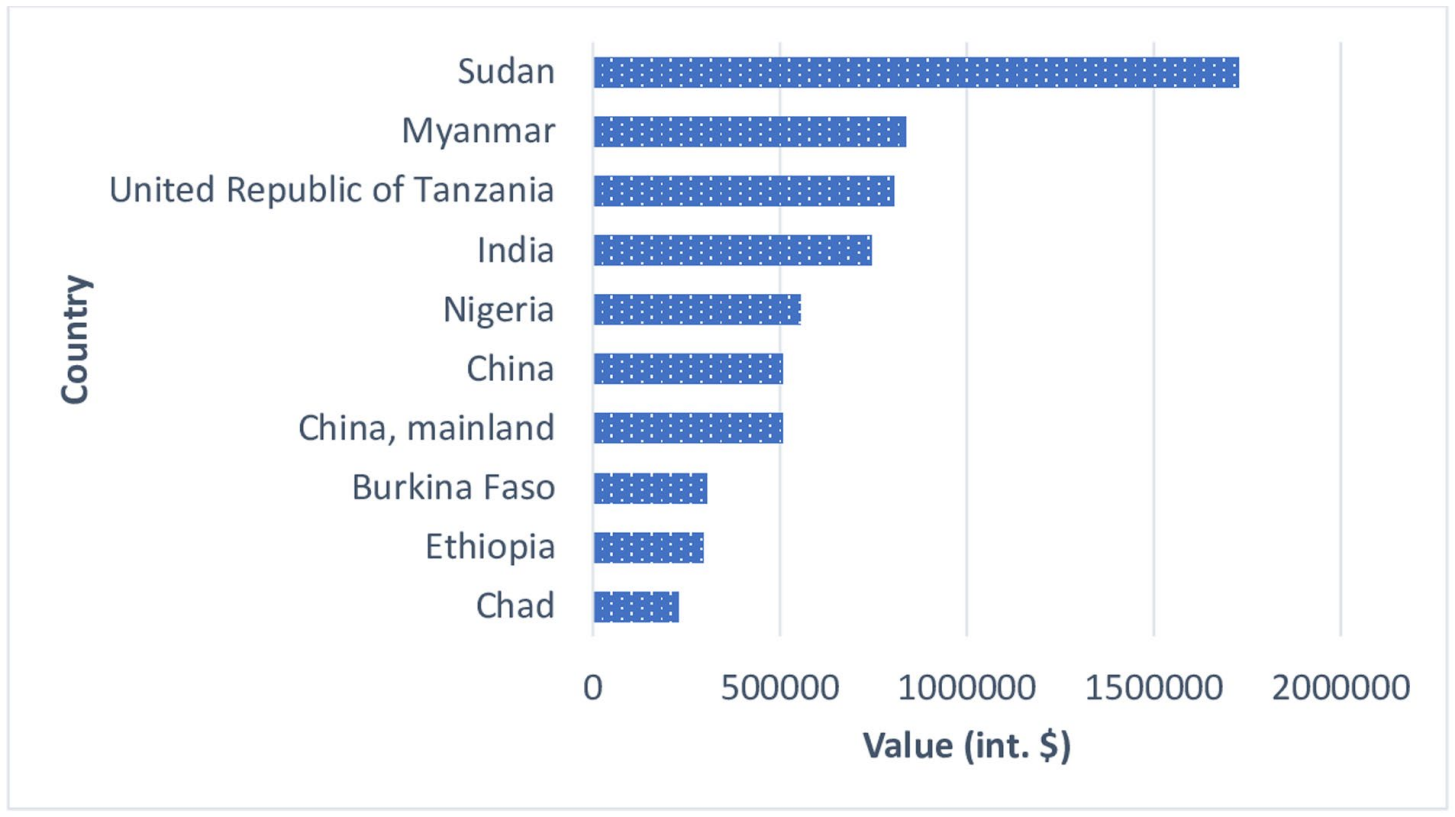
Ranking of the top 10 sesame seed producers in the world (9).

The world production of sesame seeds, as estimated by FAO (9), was 7,706,642 tons in 2020. This is 3.89% more than the previous year and 57.37% more than ‘10 years ago. Historically, total sesame seed production reached a record high of 7,706,642 tons in 2019 and an all-time low of 1,419,988 tons in 1961. The average annual growth has been 2.67% since 1961. The top 10 producers are Sudan (1,727,474 tons), Myanmar (838,193 tons), Tanzania (804,212 tons), India (745,312 tons), Nigeria (555,019 tons), China (509,091 tons), China mainland (506,515 tons), Burkina Faso (305,827 tons), Ethiopia (294,792 tons), Chad (228,888 tons) (Figure 1). The top-ranked country, Sudan, accounts for 22.42% of the world’s sesame seed production. The top 3 countries hold a share of 30.98% whiles the 10 largest countries hold around 84.54% in 2020 (9). Africa collectively accounts for over 62.95% of the sesame production in 2020 and over the past 10 years, it has established itself as a fast-growing supplier of sesame seeds in the global market. Based on a comparison of 72 countries in 2020, Burkina Faso ranked eighth in sesame seed production with 305,827 tons (9). China is the main world importer of sesame with almost 70% of world purchases. In addition, overall, observers point out that this increase in Chinese imports reflects the strength of consumption which, in the end, well resisted the coronavirus pandemic despite the uncertainties in the market from the second quarter of 2020. According to estimates, the sesame market could be worth more than $15 billion by 2025 with the growing use of the seed in the food sector as well as in the cosmetics industry.

### 2.2. Sesame seed oil production statistics

**Figure 2:**
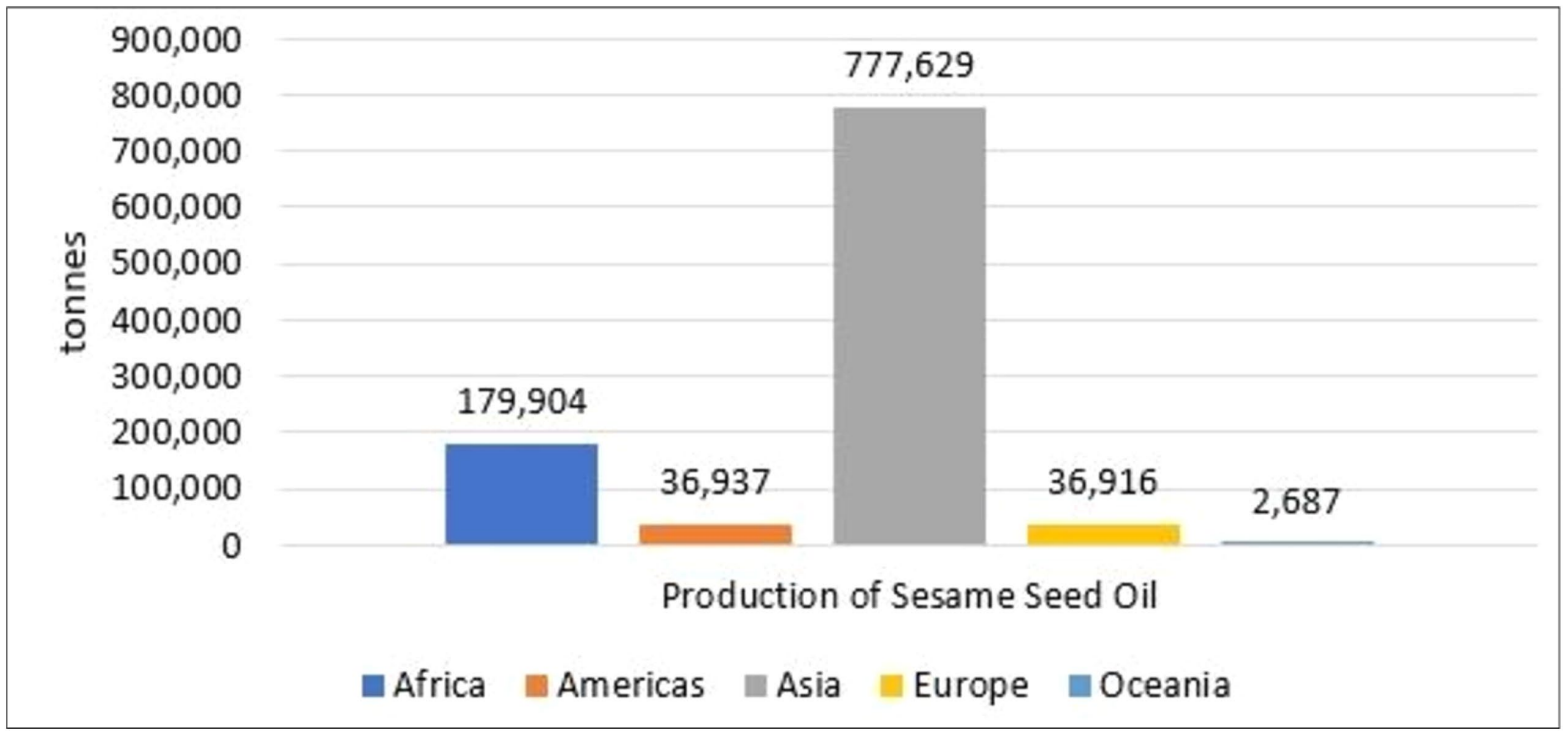
Production of sesame seed oil based on continent in 2019 (11).

The sesame seed oil (SSO) market has grown significantly in recent years, and this trend is also expected to continue during the forecast period 2022–2032. It is widely consumed because of its multiple health benefits. According to 2019 data, there are ten major producers of SSO in the World (10). These countries produce a large amount of sesame seeds. Asia was the largest sesame oil producer in 2019 with production of 777,000 tons (Figure 2) (11). The African region ranks second with a production of 179,000 tons and Europe, America and Oceania are the third, fourth and fifth largest producers with a production of 36.91, 36.93 and 2.68 tons, respectively (Figure 2). Asia and Africa collectively account for over 75 and 17%, respectively.

**Figure 3:**
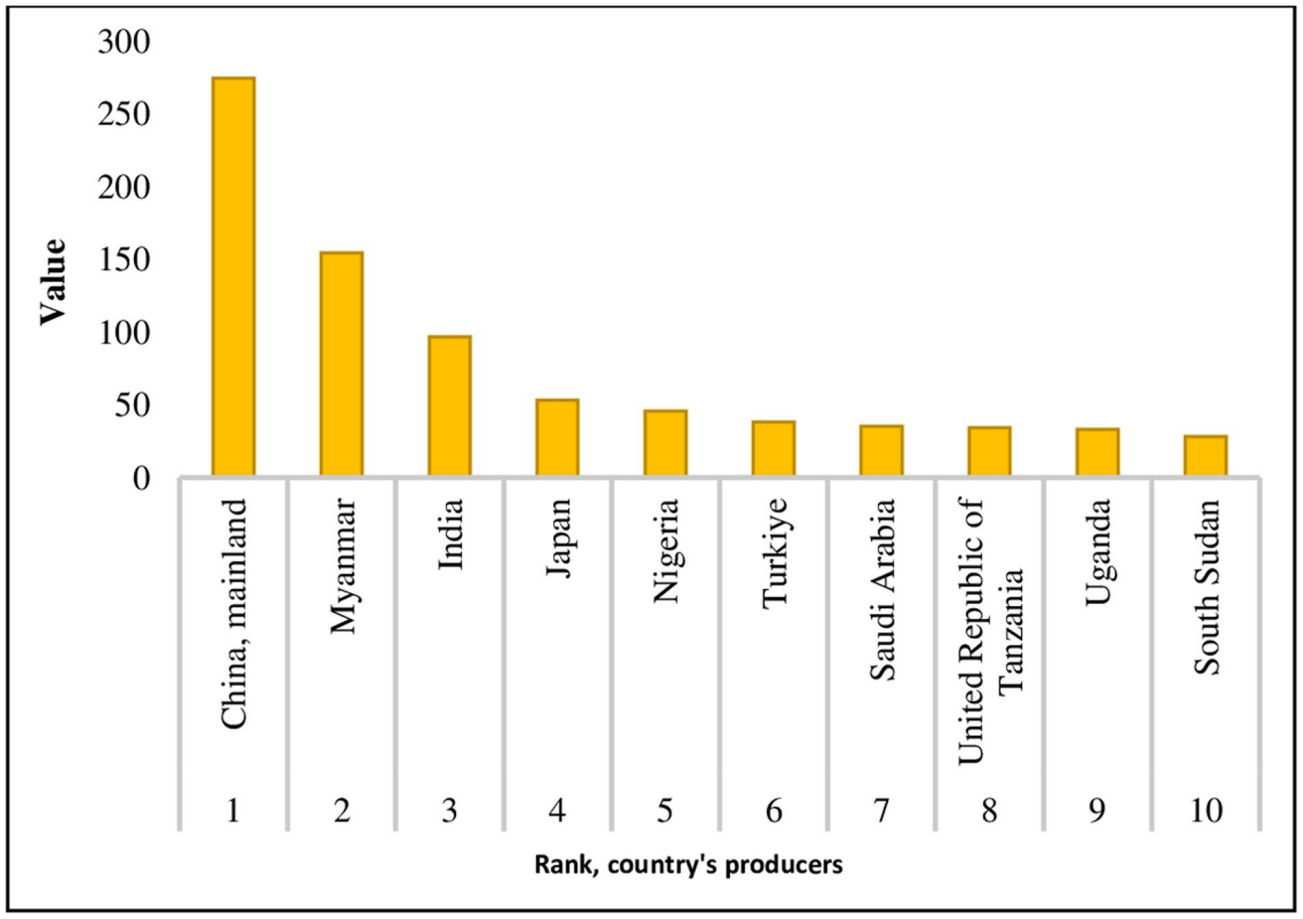
Sesame seed oil production of the top ten producing countries in 2019 (10).

During the period of 2017–2021, the sesame oil market recorded a compound annual growth rate based on value (CAGR) of 4.3%. The rank of world’s biggest SSO producers is shown in Figure 3.

## 3. Nutritional and phytochemical profile of SSO

### 3.1. Physicochemical characteristics of SSO

**Figure 4:**
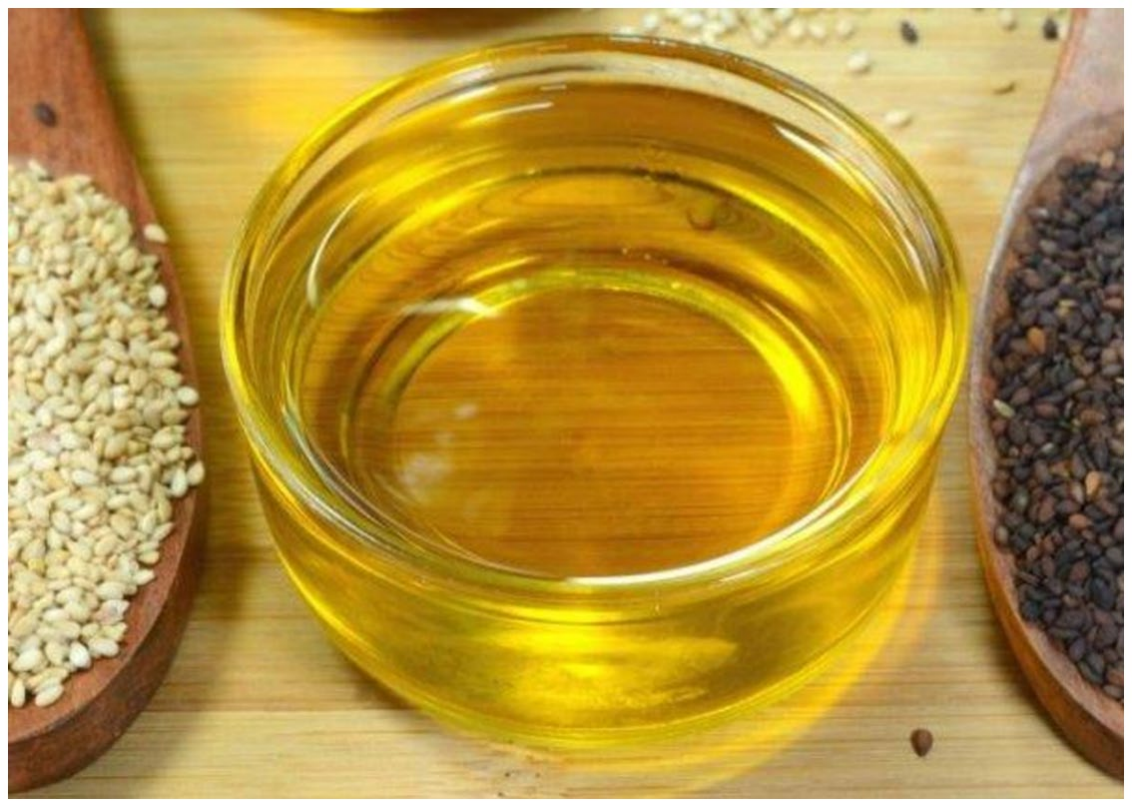
Sesame seed oil (16).

The Figure 4 and Table 1 show the SSO and the phytochemical profile of SSO, respectively. The color of SSO can be yellow or black depending on the variety (12). For color, the intensity varies from 5.8 to 8.3 Lovibond unit yellow (LUY) (13). The color of the oil is strongly affected by the roasting temperature (7). The color of SSOs is light yellow for unroasted sesame seed and brown and dark brown roasted seeds. The brightness value (L*) ranged from 43.35 to 48.66, a* color parameter from −3.97 to 1.80 and b* color parameter from 13.33 to 18.2 (7).

**Table 1:** Physicochemical characteristics of sesame seed oil.

Parameters	Saudi Arabia (7)	Republic of Congo (14)	Brazil (15)
Fat content (%)	35–62.5	49.1 ± 2.0	56.50 ± 0.67
Iodine index (g/100 g oil)	103.17–113.11	111 ± 7.5	90.17 ± 0.75
Saponification index (mg KOH/100 g oil)	185.45–200.05	186 ± 4.0	416.78 ± 1.07
Specific gravity g/cm3	0.923–0.919	-	
Unsaponifiable	-	1.93	-
Peroxide index (meqO2/kg)	-	< 0.1	-
Refractive index	1.461–1.476	-	1.465 ± 0.01
Parameter L*	43.35–48.66	-	-
a*	−3.97 -1.80	-	-
b*	13.33–18.2	-	-

As well known for other plant seeds, content levels of oils are varietal, agro-ecological and pedological dependent. Levels of lipids (organic oils) in sesame range from 35 to 62% (w/w, dry matter basis) (7, 14, 15). Physicochemical analysis of SSO indicated an unsaponifiable index content of ≈1.93%, which gives it a potential long-term conservation character (14). From nutritional aspects, low peroxide index (< 0.1 meq O_2_/kg) and the high iodine index reflect its unsaturated nature which are good quality criteria for edible oils (14).

### 3.2. Composition of fatty acid in SSO

SSO is characterized by a good balance in oleic-linoleic acid (Table 2). The presence of polyunsaturated ω-3 and polyunsaturated ω-6 fatty acids allows to categorize SSO of n-oleic/linoleic oil (40/44.1%). It contains less than 20% of saturated fatty acids, mainly palmitic acid (7.9–12%) and stearic acid (4.8–6.1%), while oleic and linoleic acids constitute more than 80% of the total fatty acids of this oil. The fatty acid profile indicates interesting proportions of monounsaturated fatty acids (40.3%), polyunsaturated fatty acids (PUFAs) (44.4%) or 44.1% acid linoleic and 03% acid linolenic (14). The ω-3 and ω-6 fatty acids are precursors of eicosanoids that regulate the immune system and inflammatory functions. Certain essential fatty acid (EFA) derivatives, such as arachidonic acid and dihomo-gamma linolenic acid, both of the ω-6 series, and eicosapentaenoic acid, of the ω-3 series, are also present in SSO. They are of great importance because they are lipid mediators involved in many physiological functions (17).

**Table 2:** Composition of fatty acid in sesame seed oil.

Fatty acid profile of sesame oil	Republic of Congo e (%) (14)	Brazil (%) (18)	Brazil (%) (15)	Iran (%) (19)	Saudi Arabia (%) (7)
Saturated fatty acids					
Caprylic (C8:0)	-	-	-	-	3.22 ± 0.27
Capric (C10:0)	-	-	-	-	2.06 ± 0.12
Lauric (C12:0)	-	-	-	-	14.59 ± 0.49
Acid myristic 14: 0	<0.1	0.02 ± 0.00	0.04 ± 0	-	4.38 ± 0.13
Acid palmitic 16: 0	9.2	9.21 ± 0.08	11.49 ± 0.04	10.7 ± 0.2	7.49 ± 0.04
Acid margaric 17: 0	0.1	0.07 ± 0.00	0.05 ± 0.00	-	0.03 ± 0.02
Acid stearic 18: 0	5.2	6.06 ± 0.04	2.64 ± 0.01	6.5 ± 0.4	4.04 ± 0.06
Acid arachidonic 20: 0	0.6	0.66 ± 0.01	0.52 ± 0.01	0.25 ± 0.2	0.35 ± 0.01
Acid behenic 22: 0	0.1	0.13 ± 0.00	0.23 ± 0.01	-	0.08 ± 0.01
Acid lignoceric 24: 0	0.1	0.09 ± 0.00	0.22 ± 0.00	-	0.11 ± 0.01
Ticosanic (C23:0)	-	-	0.04 ± 0.00	-	-
Monounsaturated fatty acids					
Acid palmitoleic 16: 1	0.1	-	0.14 ± 0.01	-	0.08 ± 0.01
Acid erucic 22: 1	<0.1	-	0.02 ± 0.00	-	-
Acid nervous 24: 1	<0.1	0.1 ± 0.00	-	-	-
Acid oleic 18: 1	40.0	-	35.32 ± 0.27	41.8 ± 0.2	28.59 ± 0.44
Polyunsaturated fatty acids					
Acid linoleic 18: 2	44.1	-	47.62 ± 0.19	40.09 ± 0.05	28.35 ± 0.46
Acid linolenic 18: 3	0.3	-	1.25 ± 0.05	0.8 ± 0.2	0.29 ± 0.01
Acid Gondoic/Eicosenoic 20: 1	0.2	-	0.25 ± 0.01	-	0.05 ± 0.01
Cys-eicosadienoic (C20:2)	-	-	0.01 ± 0.01	-	-

### 3.3. Lignans

Lignans are a group of naturally occurring compounds that are defined as an oxidative coupling product of β-hydroxyphenylpropane and widely distributed as minor compounds in the plant kingdom. Although SSO contains nearly 85% unsaturated fatty acids, it is known to be highly resistant to oxidative rancidity and can be stored for long time (18–21). This specific stability is not only attributed to the presence of tocopherols, but is also associated with lignans (2, 18–20, 22, 23). The main SSO lignans are: sesamin (344.19–393.25 mg/100 g), sesamolin (147.12–202.92 mg/100 g) and sesamol (70,00–610,00 mg/100 g) (Table 3). Their content depends on the variety. Black seeds contain less oil and a high sesamolin to sesamin ratio (24, 25).

**Table 3:** γ-tocopherol, lignan (sesamin, sesamolin), total phenolic compounds (tpc) and composition of sterols in sesame seed oil.

Parameters (%)	Turkey (31)	Saudi Arabia (7)
γ-tocopherol (mg/100 g)	-	23.18–25.93
Sesamolin (mg/100 g)	-	147.12–202.92
Sesamin (mg/100 g)	-	344.19–393.25
Total phenolic (mg gallic Acid equivalent per 100 g)	-	152.2–193.87
Sterols (%)		
Campesterol	17.28–21.99	31.38–34.06
Stigmasterol	4.73–5.99	5.1–5.2
β-Sitosterol	61.19–67.60	48.42–50.55
Δ5-Avenasterol	6.57–10.25	10.68–11.78
Δ7-Stigmasterol	1.38–3.12	0.88–0.99
∆-7-Avenasterol	-	0.14–0.22
Total sterols (mg/100 g)	461,2.–535,26	740.2–896.4

### 3.4. Tocopherols

Tocopherols (vitamin E) refer to a set of molecules containing 2-methyl-6-chromanol ring and a fully saturated phytyl chain (26). The carbon chain exists in two forms: a form comprising three un-saturations which characterize tocotrienols and a totally saturated form characterizing tocopherols. The different forms of tocopherols (α, β, γ and δ) are distinguished from each other by the number and location of the methyl groups attached to the chromanol ring (27). SSO is well known for its oxidative stability; one of the reasons for this stability is attributed to its tocopherol content which ranged from 33 mg/100 g to 101 mg/100 g according to the Codex Alimentarius Standard (28). SSO obtained from black seeds contains less tocopherols than that obtained from brown or white seeds. Jiang et al. (29) find that γ-tocopherol, the most abundant form in sesame seeds, may be important to human health, compared to α-tocopherol, the predominant form of vitamin E in humans and animals tissues and the main form of supplements. Indeed, γ-tocopherol seems to be a trap more effective for lipophilic electrophiles than is α-tocopherols. The γ-tocopherol content of SSO ranges from 23.18 to 25.93 mg/100 g (Table 3).

### 3.5. Phytosterols, phylloquinone and total phenolic

Phytosterols or plant sterols are essential components of membranes, playing an important role in controlling membrane fluidity and permeability as well as in signal transduction. Their role in plant cells is similar to that of cholesterol in mammalian cells (30). Sesame seed oil (SSO) is one of the richest sources of phytosterols (740.2–896.4 mg/100 g) (7). The phytosterols include campesterol, stigmasterol, ∆5-avenasterol, ∆7-avenasterol, ∆7-stigmasterol, and β-Sitosterol which is the most abundant sterol in SSO (31). SSO contains phylloquinone (vitamin K) (13.6 mg/100 g) and total phenolic (152.2–193.87 mg gallic Acid equivalent per 100 g) (7). Table 3 represent the composition of sterols and total phenolic in SSO.

### 3.6. Nutritional quality indexes

Nutritional quality of sesame seed lipid fractions was evaluated by different indexes as presented in Table 4. The PUFA: saturated fatty acids (SFA) ratio of 0.79 in SSO indicates that this oil has a good balance of fatty acids (15). The determination of the ω6: ω3 ratio is important for human health because a consumption of ω6, accompanied by a decrease in the ingestion of ω3, is a risk factor for cardiovascular disorders. These fatty acids compete for enzymes involved in desaturation reactions and chain elongation. Although these enzymes have a higher affinity for ω-3 fatty acids, the conversion of linolenic acid into long-chain PUFAs is strongly affected by dietary levels of linolenic acid (32). The ω6/ω3 ratio of SSO are ≈97. The AI (pro-and anti-atherogenic) and TI (pro-and anti-thrombogenic) indices are related to risk factors for cardiovascular disease. Thus, the values of these indices must be kept low. The AI nd TI were less than 1 for SSO, due to the cardioprotective effect of their PUFAs.

**Table 4:** Evaluation of SSOs by nutritional quality indexes.

INDEX	SSO	Reference
PUFA:SFA ratio	0.79	
ω6: ω3 ratio	97.80	
Atherogenicity index (AI)	0.69	
Thrombogenicity index (TI)	0.13	(13)
Hypocholesterolemic: hypercholesterolemic ratio (HH)	4.82	

The nutritional value of SSO is directly linked to the four main physiological roles of lipids: source of energy (1 g of lipid = 9 kcal); important structural role as constituents of cell membranes; precursors of molecules with high biological activity or “chemical oxygen mediators” playing an important role in vital functions (platelet aggregation and blood coagulation, renal function, inflammatory and immune phenomena, skin aging, etc.) and the supply and carrier of fat-soluble vitamins (A, D, E and K) (33).

## 4. Comparison of SSO with other vegetable oils

**Figure 5:**
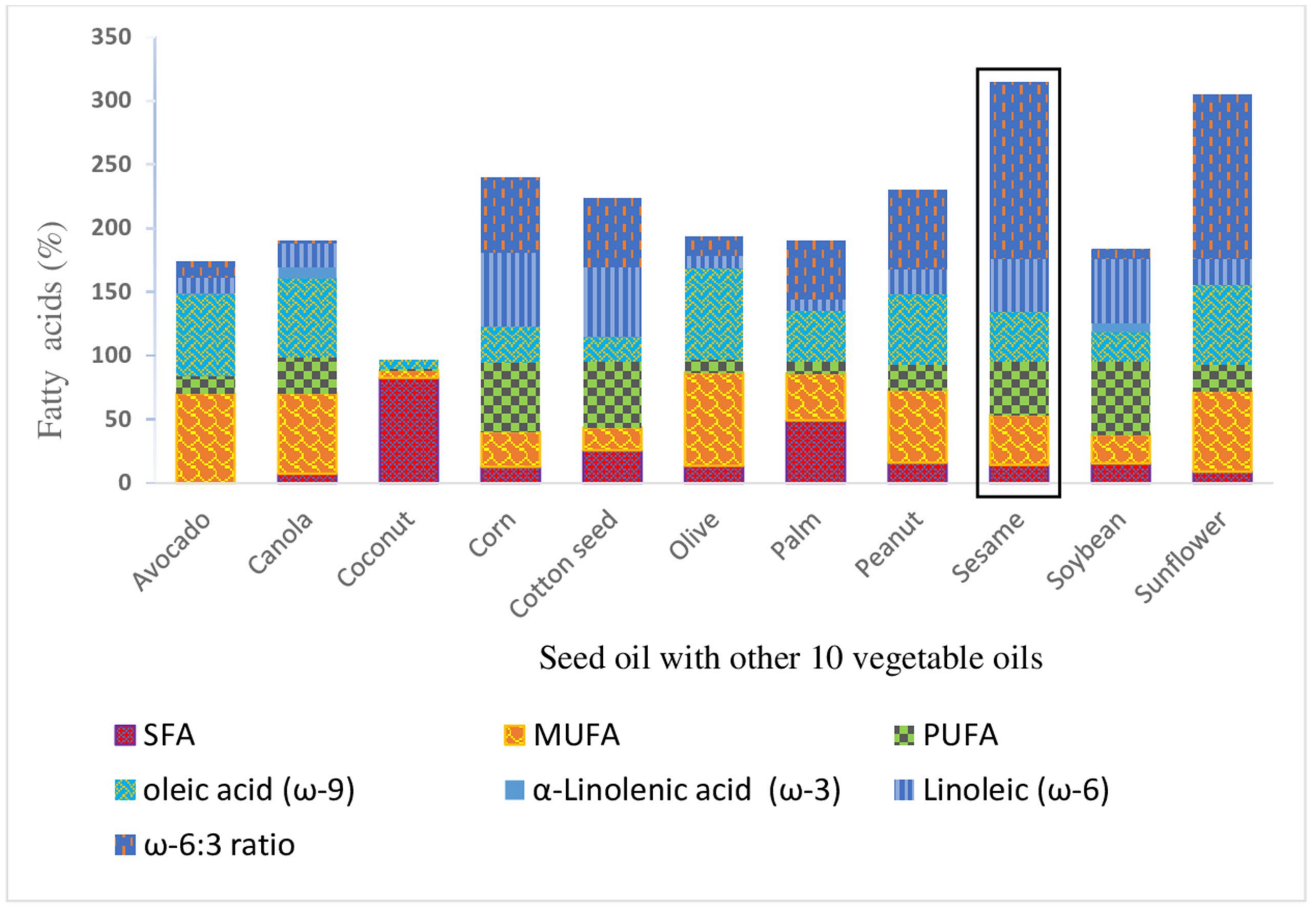
Comparison of sesame seed oil with other 10 vegetable oils (35).

SSO is an oleic/linoleic oil compared to other vegetable oils (Figure 5) Oleic acid and linoleic acid constitute more than 80% of the total fatty acids with percentages of 39.3% for oleic acid and 41.3% for linoleic acid (34, 35). SSO is one of the most stable edible oils despite its high degree of unsaturation. Depending on the fatty acid composition, fatty substances of plant origin are divided into different families. They may belong to the following families (33):

- oleic where this fatty acid, main representative of monounsaturated fatty acids (MUFA), is predominant: olive, peanut, hazelnut oils, sunflower and rapeseed varieties rich in oleic acid and rapeseed oil;
- linoleic where this fatty acid (C18:2, omega-6), polyunsaturated fatty acid (PUFA), is predominant: soybean, sunflower, corn germ and grape seed oils;
- α-linolenic where this fatty acid (C18:3 omega-3/PUFA) is present in significant quantities: rapeseed, soybean, walnut and flaxseed oils where this fatty acid is predominant;
- fatty substances rich in saturated fatty acids (SFA) with their main representatives (C12:0, C16:0, C18:0) present in medium to high quantity: palm oils, palm kernel oils and rich copra in lauric acid (C12:0), cocoa butter and for comparison butterfat;
- α-linolenic other than flax, distinguished from each other by their major fatty acid: oleic acid for rapeseed oil and linoleic acid for soybean and walnut oils.

SSO contains almost as much oleic acid as linoleic acid. Oils belonging to oleic, linoleic and α-linolenic families contain an average of 8–15% SFA, with peanut exceeding this range with around 20% (or more).

The temperature at which the oil begins to smoke, break down, form a bluish smoke and be damaged in flavor and nutritionally altered is called melting temperature. The smoke point of SSO is between 177°C (unrefined) and 232°C (refined), less than olive oil (190°C unrefined to 242°C, refined) (36). The higher this value, the less the fatty acids that make up the oil can degrade and therefore alter the smell and taste of the dish. Frying requires oil with a high smoke point, usually around 175–190°C (37). Refined oils have a considerably higher smoke point because they have been stripped of vitamins and protection by phytochemicals (37). The high PUFA oils were never really considered suitable for high heat. The most suitable are oils with high levels in MUFA with some content of linoleic acid (for flavor) and saturated fatty acids for stability (38).

The presence of natural lignan-like antioxidants (sesamin, sesamolin and their derived-products, sesamol and sesaminol, respectively) gives the oil superior stability and beneficial physiological effects. These lignans are more specific to SSO, they are not found in many vegetable oils. SSO is one of the richest sources of phytosterols. SSO stored with several types of vegetable oils at 60°C in an open container remains stable after 50 days. A rapid increase in oxidative degradation was noticed after 10 days for the other vegetable oils. This antioxidant character can be explained by the presence of several active molecules such as sesamol, γ-tocopherol and sesamin. This later constituent exerts its antioxidant power in several ways. In fact, sesamin has effects on hypertension, atherosclerosis, thrombosis, obesity and diabetes by multiple routes. The main source of these effects is the antioxidant and anti-inflammatory properties of sesamin (39). By inhibiting the production of reactive oxygen species (ROS), sesamin enhances nitric oxide (NO) bioactivity in blood vessels, therefore may reduce endothelial dysfunction and hypertension, decreasing vascular inflammatory response, and alter the progression of atherosclerotic lesion formation and thrombosis. Sesamin can also impede the development of type-II diabetes by protecting pancreatic β-cells (39). Sesamin also regulates adipogenesis and obesity by inhibiting the absorption of fat from the gastrointestinal tract, increasing the activity of lipolytic enzymes, decreasing the activity of lipogenic enzymes, preventing the differentiation of preadipocytes into mature adipocytes, inducing apoptosis in mature adipocytes, and reducing lipid droplets in mature adipocytes (39).

## 5. Antioxidant potential

Phytosterols, tocopherols (vitamin E), and lignans present in SSO are powerful antioxidants (Table 5) (40). These compounds scavenge free radicals in the body to reduce the risk of developing chronic diseases. SSO is an excellent source of PUFAs and exhibit cardioprotective properties. It displays good oxidative stability due to its high content of these fatty acids. SSO brute contains an negligibleamount of sesamol, but it has antioxidant properties. Sesaminol, appears to display antioxidant activity comparable to that from sesamol in non-heated oil. SSO naturally contains vitamin E, an antioxidant that protects cell membranes from oxidation. It protects the skin from external aggressions such as UV radiation. The active principles sesamin, sesamolin, sesamol, sesaminol are other natural antioxidants interesting for the reconstitution of cells. The presence of these two unusual constituents in the oil, *e.i* sesamin and sesamolin, gives SSO a strong antioxidant power, therefore a great stability to oxidation (41, 42). the SSO used in massage increases the elasticity of the skin and slows down the aging phenomenon.

**Table 5:** Main bioactive compounds in sesame seed oil.

Name of bioactive compound	IUPAC name molecular	Category of component	Molecular formulas	Structure	Reference
Lignans	-	Sesamin	C_20_H_18_O_6_	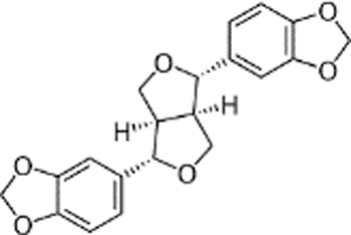	(40)
	2*H*-1,3-Benzodioxol-5-ol	Sesamol	C_7_H_6_O_3_	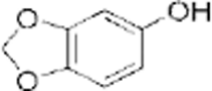	
	-	Sesamolin	C_20_H_18_O_7_	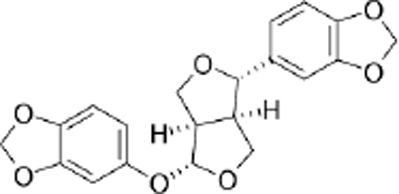	
Tocopherol	-	γ-tocopherol	C_28_H_48_O_2_	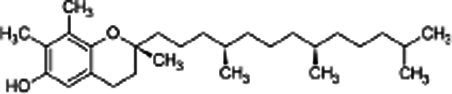	
Polyunsaturated	Hexadecanoic acid	fatty acids Palmitic acid	C_16_H_32_O_2_	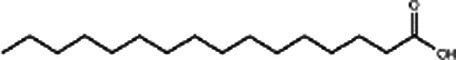	
	(9Z)-Octadec-9-enoic acid	Oleic acid	C_18_H_34_O_2_	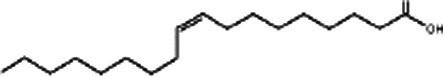	
	(9Z,12Z)-octadeca-9,12-dienoic acid Telfairic acid	Linoleic acid	C_18_H_32_O_2_	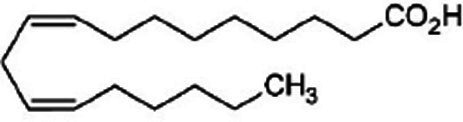	
	all-cis-9,12,15-octadecatrienoic acid	Linolenic acid	C_18_H_30_O_2_	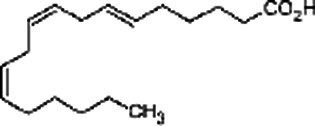	
Plant sterols	5α-Campestan-3β-ol	Campestanol	C_28_H_50_O	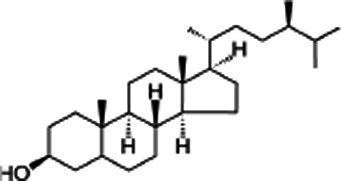	
	*5α-Stigmastan-3β-ol*	Sitostanol	C_29_H_52_O	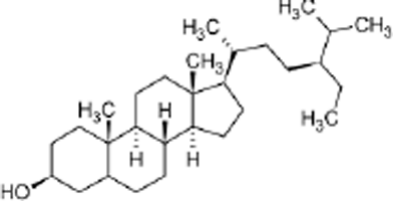	
	(3β,24Z)-Stigmasta-7,24 (28)-dien-3-ol	Avenasterol	C_29_H_48_O	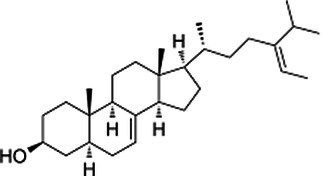	
	Stigmasta-5,22-dien-3β-ol	Stigmasterol	C_29_H_48_O	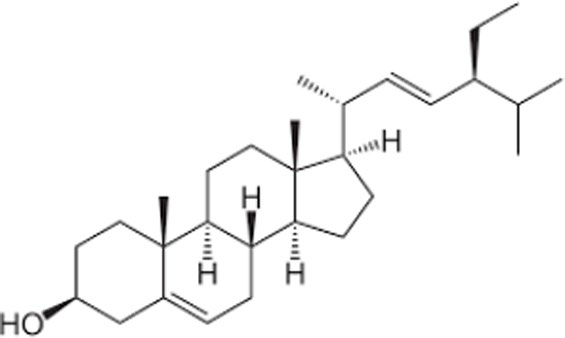	
	3beta-hydroxy-Delta (5)-steroid	Beta-sitosterol	C_29_H_50_O	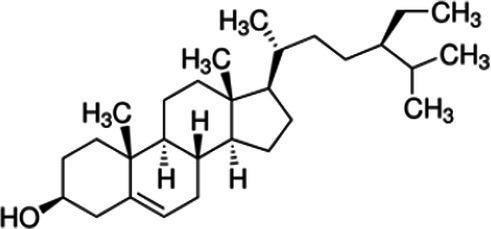	

## 6. Health benefits of SSO

### 6.1. Low-density lipoprotein cholesterol

The fatty acids of SSO have an essential place and for two of them, essential: linoleic acid and α-linolenic acid because man is unable to synthesize them; he must therefore find them in adequate quantity in his diet. These two fatty acids belong to the class of polyunsaturated fatty acids (PUFAs), each being leader of the series of PUFAs called (n-6) and (n-3), respectively. SSO is a very interesting oil at the dietary level. Its balance between oleic acid C18:1 and linoleic acid C18:2 makes this oil a very good table oil in diets aimed at preventing cardiovascular diseases and increasing “bad cholesterol.” SSO is heart-healthy and delicious because of its fatty acid content. According to research conducted on the effect of SSO, it could be able to reduce bad cholesterol levels, because it contains a wide range of PUFAs, including sesamol and sesamin. These fatty acids keep the cardiovascular system strong and LDL cholesterol levels low, thus preventing atherosclerosis. Sesamin is one of the active compounds present in SSO and justifies the antinociceptive and anti-inflammatory properties of this oil (43). This means that one is better protected against heart attack and stroke if one adds SSO to the diet. The higher fatty acids, eicosapentaenoic (EPA) and docosahexaenoic, have been shown to have a hypotriglyceridemic effect (44). Recent studies on this oil have shown the role of oleic acid in the metabolism of circulating cholesterol *via* lipoproteins (45). It leads to a decrease in LDL-cholesterol and jointly to an increase in HDL-cholesterol, which reduces the risk of atherogenesis. It has antilipolytic effects in the body and can prevent the oxidation of LDL-cholesterol (3). Its cholesterol-lowering activity is linked to phytoestrogen which increases HDL and lowers LDL, VLDL, TC and TG (3). Sesamin from SSO mainly exercises anti-hyperlipidemic effects (46). Monounsaturated fatty acids (MUFA) could play a role in the prevention of dyslipidemia and atherosclerosis (47). Given the importance of reducing saturated fatty acids in the diet.

### 6.2. Blood sugar

SSO can help regulate blood sugar. A study on diabetic adults consuming SSO showed a drop in fasting blood sugar (blood sugar after a night’s sleep) and hemoglobin A1c (average blood sugar over the past two to 3 months). Also, a study conducted by Haidari et al. (48) on diabetics showed that those treated with SSO had significantly lower glucose levels and higher high-density lipoprotein levels than the diabetic control group. Other studies have shown lower total cholesterol, triglycerides, fasting blood sugar, insulin resistance, inflammation and oxidative stress. This can be explained by the antioxidant action of the vitamin E as well as the lignans and unsaturated fatty acids contained in SSO. A synergistic effect of SSO with glibenclamide (glyburide), an hypoglycemic drug may provide a safe and effective drug combination solution that may be very useful in clinical practice for the improvement of hyperglycemia (49). SSO as a functional food may play an important role in regulating fasting blood sugar levels and against the deleterious effects of diabetes in humans with type 2 diabetes (50). It can also improve biomarkers of liver, heart and kidney function significantly in diabetics (50). The blend of 20% cold-pressed unrefined SSO and 80% physically refined rice bran oil as cooking oil, lower hyperglycemia and improve the lipid profile in type 2 diabetes mellitus patients (51).

### 6.3. Heart health

SSO has a balanced ratio of ω3, ω6, and ω9 fatty acids. Research showed that a diet containing these healthy fats reduces the risk of developing heart disease. Some studies showed that consuming SSO can help lower LDL cholesterol and triglycerides, which play a role in protecting the heart. The presence of lecithin, through its role as an emulsifier, opposes the deposit of cholesterol and saturated fats in the arteries. On the other hand, sesamin has been reported to inhibit ∆5-desaturase activity as well as cholesterol absorption; and together with the antioxidant activities of sesaminol, it has been held responsible in the prevention of cancer (52). Sesamol is a potent inhibitor of fungal fatty acid biosynthesis. This effect is apparently due to the inhibition of the malic enzyme and the supply of NADPH necessary for this biosynthetic pathway. It is sesamol’s ability to reduce the synthesis of the coenzyme, NADPH, that makes it attractive for studying the effect of oxidants on tumor and vascular endothelial cells (53).

### 6.4. Cancer

SSO contains high levels of lecithin (2.85 to 3.57%) making sesame seeds an alternative sources of lecithin (54). Lecithin is effective in reducing hepatic steatosis for patients with parenteral nutrition diseases and an effective treatment against dermatitis and dry skin. SSO contains high levels of triglyceride-bound linoleate which selectively inhibits the development of melanomas. It has been shown to inhibit the growth of malignant melanoma *in vitro* and the proliferation of human colon cancer cells (55).

In liver cancer, studies have shown that oral administration of SSO exerts significant protective effects against diethylnitrosamine (DEN) induced oxidative and hepatic damage by increasing the antioxidant defense mechanisms of the host liver (56). This could be attributed to improvement of anemia, decrease in activation of serum liver enzymes, reduced degree of hepatic vacuolation and necrosis of direct endoscopic necrosectomy (DEN) which improves cancer treatment (56). Low-calorie diets may have favorable effects for NAFLD (nonalcoholic fatty liver disease) patients by alleviating obesity and fatty liver disease. The desired effects of weight loss may be enhanced by consuming a low-calorie diet enriched with sesame seed through improvement in fatty liver severity and serum ALT (alanine aminotransferase) and AST (aspartate aminotransferase) levels (57). Application of massage with SSO as a complementary method is effective in reducing the pain severity of patients with Chemotherapy-induced phlebitis in patients with colorectal cancer (58). SSO decreased the size of cardiomyocytes and the levels of cardiac renin, angiotensin-converting enzyme and angiotensin II (58). He modulates cardiac renin-angiotensin system to ameliorate left ventricular hypertrophy by inhibiting mitogen-activated protein kinases activation and lowering oxidative stress (59).

### 6.5. Memory problems and Alzheimer’s disease

**Figure 6:**
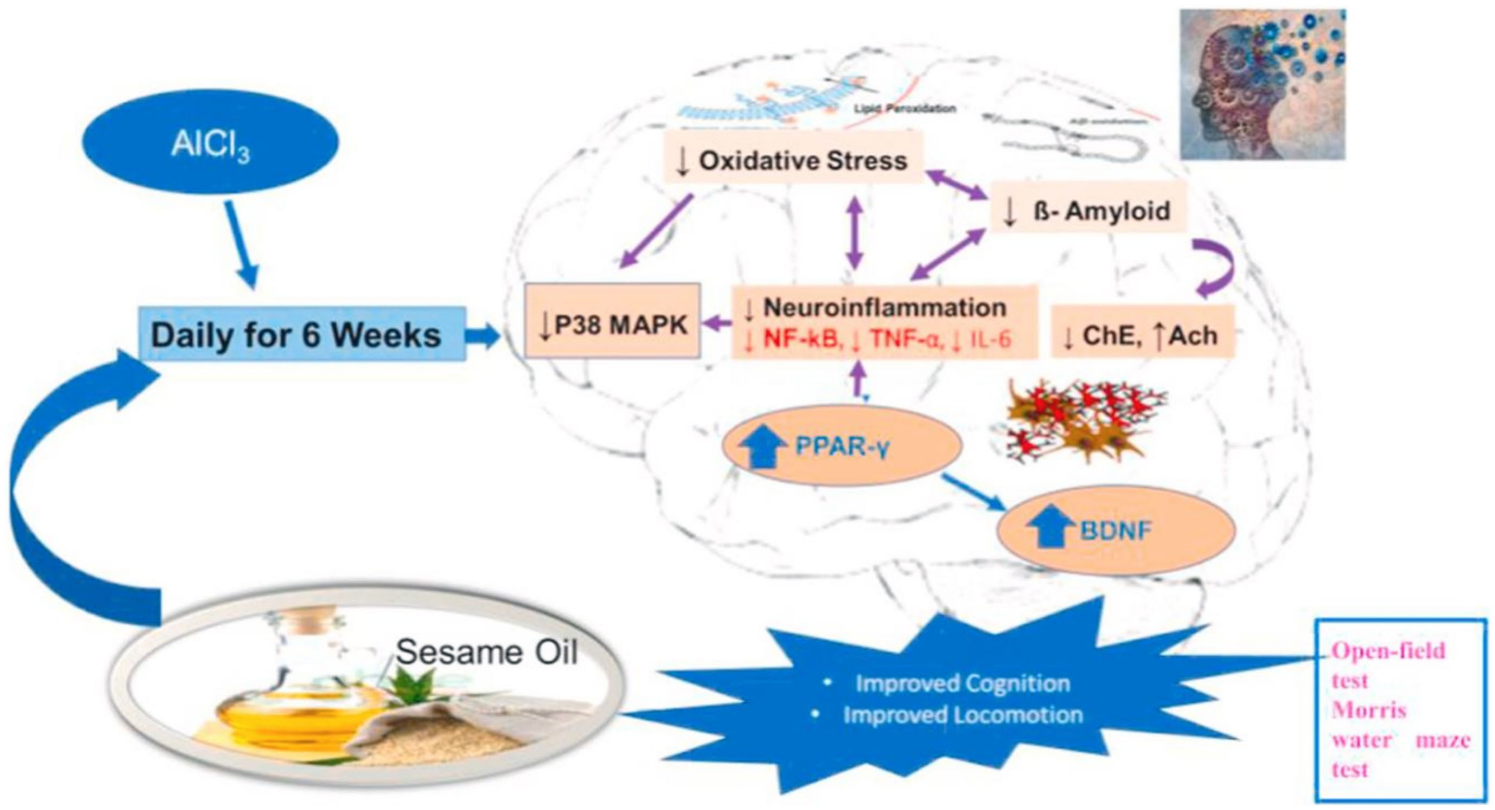
Graphical summary of the effect of sesame on memory impairment, oxidative stress and neurodegeneration in Alzheimer’s patient (60).

SSO alleviates memory impairment, oxidative stress, and neurodegeneration in Alzheimer’s patients (60). The neuroprotective effect of SSO involves the modulation of different mechanisms targeting oxidative stress, neuroinflammation and cognitive functions. SSO can modulate different molecular targets involved in the pathogenesis of Alzheimer’s disease through alterations in nuclear factor kappa B/p38 mitogen-activated protein kinase/brain-derived neurotrophic factor/peroxisome proliferator-activated receptor gamma (NF-κB/p38MAPK/BDNF/PPAR-γ) and this can be attributed to the synergistic effect of their active components (60) (Figure 6). A 12-week SSO supplementation in elderly people with memory impairment may result in improved overall cognitive function, by improving verbal learning memory function (61). Long-term consumption of sesaminol may inhibit the accumulation of pathogenic Aβ in the brain (62).

### 6.6. Reduced inflammation

Many worldwide cultures have used SSO in traditional medicine as an anti-inflammatory product. Traditional taiwanese medicine has used it to treat joint pain, a toothache, cuts, scrapes, premenstrual cramps, etc. Although more human studies are needed, some *in-vitro* studies sugges tthat SSO is effective in lowering inflammatory markers. Deme et al. (63) showed that pretreatment with SSO could delay/reduce atherosclerosis. Genetic analysis has shown that high-fat (HF) diet supplemented with SSO reduced the expression of genes involved in inflammation and induced those involved in cholesterol, metabolism and reverse cholesterol transport, a process anti-inflammatory. These studies have shown that a diet enriched with SSO could be an effective non-pharmacological treatment for atherosclerosis by controlling inflammation and regulating lipid metabolism (47, 64, 65). Anti-inflammatory molecules associated with SSO may contribute anti-inflammatory and anti-atherosclerotic activities (63).

### 6.7. Preventing sun damage

SSO is a useful natural UV protectant. Some research showed that the antioxidants in SSO can protect the skin from UV damage. SSO is resistant to up to 30% of UV rays, while other oils are only resistant to 20% (66). SSO is a pharmaceutical carrier used as a solvent for intramuscular injections, and has nutritive, emollient properties and has been used as a laxative. In the hypoderm tissues, this oil neutralizes oxygen radicals. It quickly penetrates the skin and enters the bloodstream through the capillaries. Research is limited on this topic. Although some sources claim that SSO can be an effective natural sunscreen.

### 6.8. Other health benefits of SSO

It has other uses for the treatment of blurred vision, vertigo and headaches. The Indians use SSO as a mouthwash, antibacterial, to relieve anxiety and insomnia. SSO is significantly more effective in treating dry nasal mucus due to a dry winter climate than isotonic sodium chloride solution (67). Results have shown new mechanistic insight into the activities of SSO and proven that sesamin, the key constituent of SSO, is responsible for its benefits related to auditory function, including the protection of auditory cells and the reversal of their deficiencies (68).

### 6.9. Summary of health benefit of SSO

**Figure 7:**
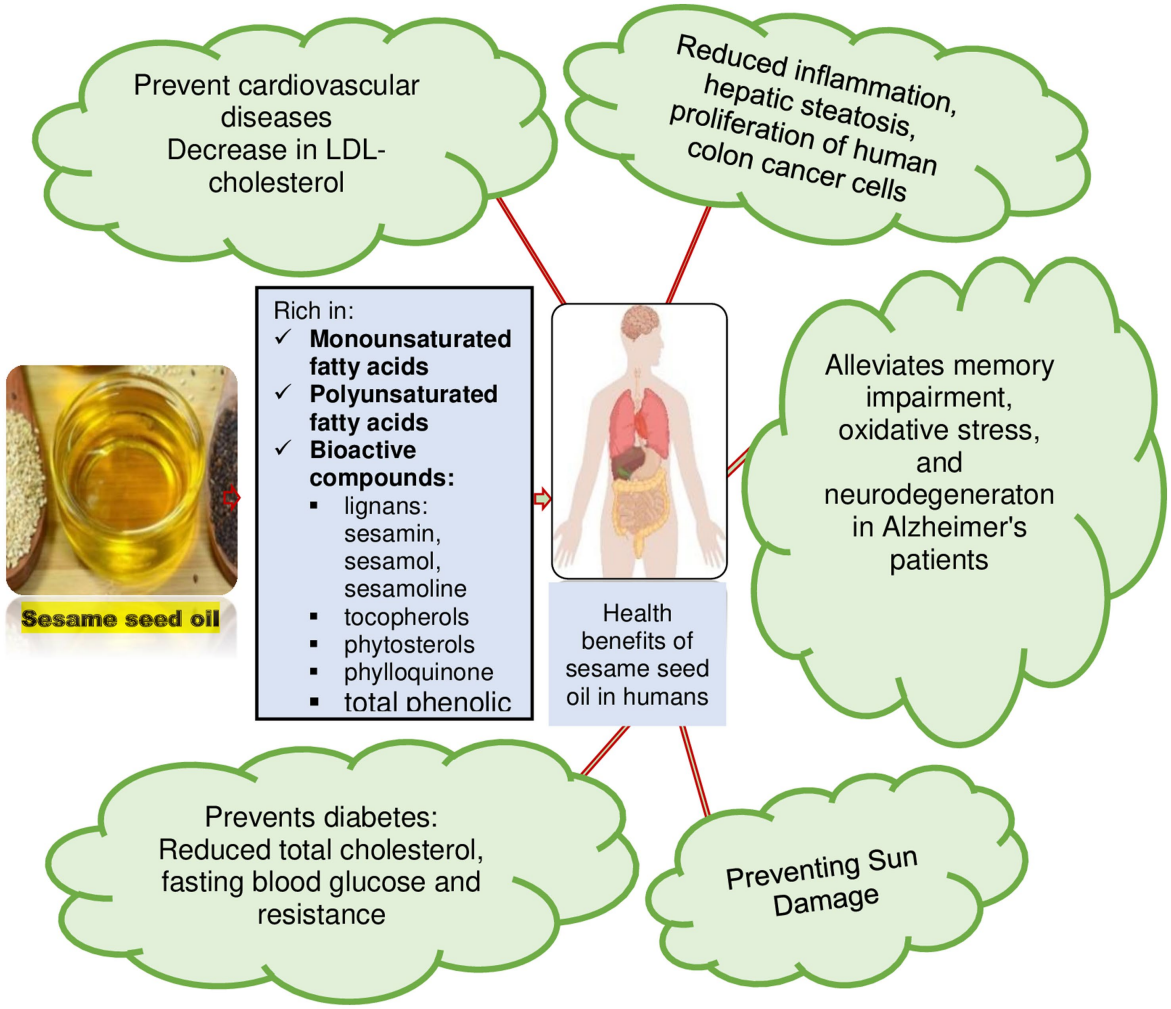
Summary of health benefit of sesame seed oil.

In summary, the benefits of SSO are associated with its richness in monounsaturated fatty acids, polyunsaturated fatty acids, bioactive compounds: lignans, tocopherols, phytosterols, phylloquinone, total phenols. Consumption of SSO is associated with reduction of diabetes, cancer, cardiovascular disease, reduction of blood pressure, reduction of bad cholesterol, reduction of inflammation, prevention of premature aging, relief of memory disorders, oxidative stress and neurodegeneration in Alzheimer’s patients (Figure 7).

## 7. Potential risks of SSO

While there are many potential benefits to incorporating SSO into the diet, there are a few potential risks to keep in mind. These risks include sesame allergies. Sesame is one of the most common allergens (69). Although this percentage may not seem significant, SSO is almost as common as the top eight allergens. Allergic reactions vary in severity, but they can be severe enough to cause anaphylaxis. Although SSO contains heart-healthy omega-3 and omega-6 fatty acids, too much oil can lead to adverse effects.

SSO is high in potential energetic value, which can lead to weight gain if consumed in excess. Fats contribute to human health, particularly that of our hair and skin, and often give good taste and flavor of meals. Some are essential and cannot be synthesized by the body. However, fatty acids, whether saturated, monounsaturated or polyunsaturated, and especially trans unsaturated (TFA), should be moderately consumed. Fats have very different properties and contributions, depending on their characteristic: cis unsaturated rather beneficial to our health, versus saturated or trans unsaturated rather unbeneficial.

On top of that, some unwanted molecules such as mycotoxins (e.g., aflatoxins and fumonisins), pesticides and trace element metals can be found in SSO (70). These molecules must not exceed the national standard range because when they exceed in a food, they can pose serious threats to human health after consumption. Transfer of aflatoxins from sesame seeds to oil may depend on the extraction technique. Traditional and cold press procedures improved 8.2 and 70.22% transfer of total aflatoxin from raw sesame to oil obtained, respectively (70).

## 8. Conclusion

Sesame seed oil (SSO) has a nutritionally rich composition in antioxidants and specific bioactive compounds such as lignans (phytosterols, tocopherols, sesamin and sesamolin, etc.). Its high antioxidant activity makes it a unique and very good quality functional food that may have positive effects on human health. Like whole seeds, extracted oil is rich in bioactive compounds. Its consumption may help to fight inflammation-related diseases such as osteoarthritis, cardiovascular diseases, neurodegenerative diseases, inflammatory bowel diseases, diabetic eye diseases, lung diseases, liver diseases, skin diseases and Alzheimer’s disease. SSO may be considered as edible oil containing high level of nutraceuticals.

## Author contributions

EBO, ZD, DC-S, and JNS developed the concept for the review, wrote manuscript and original draft preparation. RD, ZS, and FW-BT contribution to documentary research. FH-B and LTS-O contributed to the revision of the manuscript. CP and MHD provided the overall concept, revised the manuscript, and critically edited and approved the final version of the manuscript. All authors contributed to the article and approved the submitted version.

## Conflict of interest

The authors declare that the research was conducted in the absence of any commercial or financial relationships that could be construed as a potential conflict of interest.

## Publisher’s note

All claims expressed in this article are solely those of the authors and do not necessarily represent those of their affiliated organizations, or those of the publisher, the editors and the reviewers. Any product that may be evaluated in this article, or claim that may be made by its manufacturer, is not guaranteed or endorsed by the publisher.
